# Care Issues in Patients with Rett Syndrome: A Parental Perspective

**DOI:** 10.3390/children10101713

**Published:** 2023-10-21

**Authors:** Claudio Cherchi, Elena Chiappini, Maria Beatrice Chiarini Testa, Paolo Banfi, Edvige Veneselli, Renato Cutrera

**Affiliations:** 1 Pediatric Pulmonology and Cystic Fibrosis Unit, Bambino Gesù Children’s Hospital, IRCCS, 00165 Rome, Italy; mbeatrice.chiarini@opbg.net (M.B.C.T.); renato.cutrera@opbg.net (R.C.); 2 Department of Health Science, University of Florence, 50121 Florence, Italy; elena.chiappini@unifi.it; 3 Division of Pediatric Infectious Diseases Infective Diseases, Anna Meyer Children’s University Hospital, 50100 Florence, Italy; 4 Heart-Respiratory Rehabilitation Unit, IRCCS Fondazione Don Carlo Gnocchi, 20148 Milan, Italy; pabanfi@dongnocchi.it; 5 Child Neuropsychiatry, Department of Neurosciences, Rehabilitation, Ophthalmology, Genetics and Maternal and Child Health, DINOG-MI, University of Genoa, 16100 Genoa, Italy; edvige.veneselli@unige.it

**Keywords:** Rett syndrome, needs, family, survey, epilepsy

## Abstract

Background: The purpose of this study is to better understand the way caregivers of patients with Rett syndrome perceive the quality of the health care services they receive and identify its main shortcomings. Methods: A survey was distributed to all caregivers who are part of AIRETT (the Italian Association of Relatives of Patients with RS). The survey gathered information on the management of relatives of patients with Rett syndrome. Results: The data refers to 52 patients, all females, with a median age of 15 years at the time of the survey. Concerning RS specificity, our data confirm the high complexity of this chronic, multifaceted condition, mainly characterized by the presence of epilepsy, apnea, severe scoliosis, and gastrointestinal symptoms. The specialists more frequently involved in the care of patients were general practitioners or family pediatricians (98%) and neurologists (92%), and more rarely physiatrists (71%). Only 15% of patients were followed by a pulmonologist, despite the fact that respiratory problems were frequent (apneas were present in 81% of patients, and 2% had a tracheostomy). Although 63.5% of patients presented with gastrointestinal symptoms and 2% had a gastrostomy, only 33% were followed by a gastroenterologist. Moreover, although orthopedic issues were present in 78.8% of patients, including severe scoliosis in 22% of them, only 25% were followed by an orthopedist. Furthermore, despite the fact that RS patients are fragile, about one quarter of them were not vaccinated. As far as organizational issues are concerned, several specialized centers are located in various regions throughout the country. As a consequence, the high mobility rate from one center to another resulted in non-homogeneous assistance. Conclusions: The study shows that caregivers of RS patients take over most obligations and burdens by increasing their perceived level of stress. For the majority of patients, the most frequent complications were not followed by the reference subspecialist, with the only exception of epilepsy. Moreover, improving vaccination strategies for these patients is necessary.

## 1. Introduction

### 1.1. Background

Rett syndrome (RS) is a progressive gender-specific neurodevelopmental disorder occurring almost exclusively in females with an estimated prevalence of 1:10.000–15.000 live births [1,2]. Its peculiar clinical course is characterized by a normal achievement of developmental milestones from 6 to 18 months, followed by a rapid neurocognitive regression with the loss of acquired purposeful hand skills and the parallel onset of stereotypic hand movements, loss of spoken language, and ataxia [3]. The other main clinical features are microcephaly, scoliosis, cardiorespiratory dysfunction, and gastrointestinal issues, as well as respiratory and motor seizure abnormalities [4].

Most cases result from mutations in the methyl-CpG-binding protein 2 gene (MECP2), and a few atypical cases are linked to mutations in cyclin-dependent kinase-like 5 (CDKL5) and forkhead box G1 (FOXG1) [5,6]. MECP2 is abundantly expressed in brain cells, which explains the constellation of neurological symptoms and autonomic dysfunction present in this population [7,8]. Although neurological conditions are prevalent, the disease affects not only the central nervous system (CNS) but also a wide array of non-neurological organs.

RS has a complex and multifaceted clinical appearance [9]. This pathology evolves throughout the patient’s life span; multi-system comorbidities, like gastrointestinal (GI), orthopaedic, endocrine, or cardiac issues, are more or less prevalent [3]. Liver injury, urological dysfunction, adipose tissue disorders, and inflammatory response troubles are also non-neurological dysfunctions associated with RS [10]. One typical feature of RS is breathing disturbances, a well-known phenomenon classically described during wakefulness and characterized by bouts of hyperventilation followed by apnea and desaturation, breath-holding spells, and air swallowing [10,11,12,13]. Many of these chronic health issues impair the quality of life of patients with RS [14].

The aim of treatments is not only to extend the patient’s life but also to improve their well-being and independence in daily activities. Management of children with RS requires an interdisciplinary approach and should always be carried out in cooperation with the patients’ families. Therefore, chronic and multifaceted conditions require that patients often refer to more specialized centers, as also recommended by experts in recent consensus documents [2]. These structures are often far from patients’ residences and not always connected to each other. These difficulties can affect both the level of care received and the quality of life of patients and their caregivers. For this reason, it is important and necessary to direct attention not only to the kind of disorder but also to the impact that it has on the child, parents, relatives, and the entire family as a whole [15]. The stress level of parents/caregivers of children with different types of neurodevelopmental disorders (NDDs) has been recently reported in several studies that show that they experience more parenting stress than parents of normally developing children. Craig et al. [16] suggest that IQ level or emotional and behavioral problems are associated with higher levels of parenting stress. This study shows that parents/caregivers of children with NDDs should be provided with interventions and resources to empower them with knowledge and skills to reduce their stress and enhance their quality of life. Previous research on children with RS has mainly focused on the causes and treatment of the illness [15]. Although the lives of caregivers of patients with RS are centered on the process of care, current literature reports that their perceived levels of stress have been rarely investigated. Parisi et al. [15] showed that RS has a considerable impact on both the child’s development and the entire family. In this study, parents’ answers demonstrated that their child’s illness had consequences for the patient and for how the family coped with it. Other authors previously documented high stress levels in 39–44% of caregivers in relation to disease severity [17]. In a qualitative study to identify domains of quality of life for caregivers of individuals with RS, Epstein et al. [18] identified areas of quality of life as health, well-being, and pain or discomfort. The results of another survey conducted on caregivers of individuals with RS suggest that pain is a substantial concern among caregivers, particularly due to gastrointestinal and musculoskeletal conditions [19]. A qualitative study [20] explored what caregivers of individuals with RS think about the effect of this pathology on their lives, their worries about their children, and the desired improvements of the condition. Moreover, according to other studies, mothers giving care to children with RS are at high risk of severe depression.

Therefore, given the extensive assistance needs associated with RS, it is important to understand how families perceive their relatives’ primary care to improve the quality of life.

As far as we know, the current literature does not report any study investigating the caregivers’ perceptions regarding the management of the clinical problems of their relatives with RS.

The purpose of this study is to better understand the way caregivers of patients with RS perceive the quality of the health care services they receive and identify its main shortcomings. Our study focuses on the burden of responsibilities and level of stress as perceived by caregivers, the need to seek assistance in several centers, and the non-homogeneous level of assistance received in different centers.

### 1.2. Methods

A cross-sectional study was conducted, including both quantitative and qualitative approaches. Between January and March 2022, all families who are part of AIRETT (the Italian Association of Relatives of Patients with RS) were considered eligible to participate in this study. The inclusion criteria for all participants were as follows: (1) parents/caregivers of patients diagnosed with RS; (2) patients followed by an Italian center. All participants received an invitation to take part in the survey through an email with an explanation of the study scope and design and a link to answer an anonymous questionnaire. In the invitation, parents/caregivers were explained that questions referred to the management of RS children in order to focus on their unmet needs. In this first stage, the questionnaire was developed in Italian. The parents/caregivers had to be able to read and speak Italian. Each questionnaire was filled out anonymously by the parents/caregivers after reading the information on privacy regulations in accordance with European Union Regulation number 79/2016 [21] and obtaining consent to the processing of data necessary for the study.

The study was approved by the Institutional Review Board at Bambino Gesù Children’s Research Hospital.

#### 1.2.1. Survey Instrument

The survey was developed by four Italian specialists working in academic institutions, hospitals, or community settings with ≥10 years of clinical experience in the management of patients with RS (RC, pediatric pneumologist; BP, pneumologist; MBCT, pediatric pneumologist; VE, neuropsychiatrist). Questions were formulated on the basis of available literature data on the management and outcomes of patients with RS, taking into consideration, in particular, a recent U.S. Consensus Document regarding the management of RS across the lifespan [2].

A non-technical language suitable for the general population was used. Non-Italian caregivers were asked whether they were able to read and write in Italian. In cases of a negative response, a translator was asked to help.

The final questionnaire was composed of four main sections to gather the following information: (1) anagraphic and genetic characteristics; (2) clinical features; (3) type of medical interventions and regular subspecialist check-ups (i.e., cardiologic, pneumologist, neurologic visits), and immunizations; (4) therapeutic interventions, including use of drugs, physiotherapy, speech therapy, surgical interventions, and devices (i.e., percutaneous gastrostomy).

The full questionnaire is reported in Appendix A.

#### 1.2.2. Data Analysis and Statistics

All answers were automatically collected into an electronic database and then transferred into an Excel spreadsheet. Quantitative data were presented as median and interquartile range, or absolute frequency and percentage, as appropriate. When needed, 95% confidence intervals (95% CIs) were calculated. The study was funded by the AIRETT Association.

## 2. Results

A total of 52 out of 250 (26%) caregivers submitted the questionnaire. Data referring to 52 patients (Table 1), with a median age at the time of the survey of 15 years (interquartile range 7–22 years; minimum age 2 years, maximum age 44 years), were included.

Mutation type was known in 51/52 (98.1%); 43 patients (82.7%) presented the MECP2 classic RS, 6 (11.5%) the congenital variant, and 3 (5.8%) the preserved language variant.

**Figure 1:**
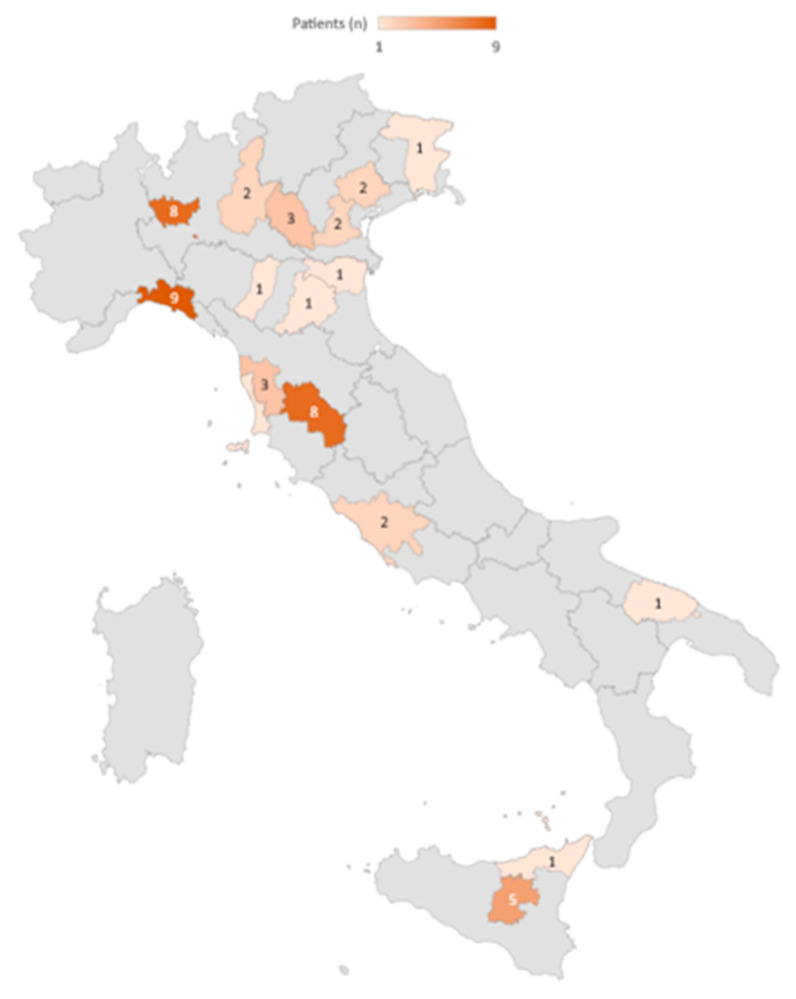
Geographical distribution of patients by clinical center.

Some caregivers reported that their relatives were followed by more than one center, and one caregiver declared that they were not followed by any center; only four centers followed five or more patients. (Figure 1).

Specialists who mainly followed these patients were neurologists/neuropsychiatrists (48; 92.3%), and in most cases, on a regular basis (28; 58.3%).

Almost all patients were followed by the general practitioner or family pediatrician; however, in the majority of cases (34/52; 65.4%), assistance was provided only on demand. Other specialists (orthopedist, pulmonologist, physiotherapist, and speech therapist) were only rarely involved (Table 2).

**Figure 2:**
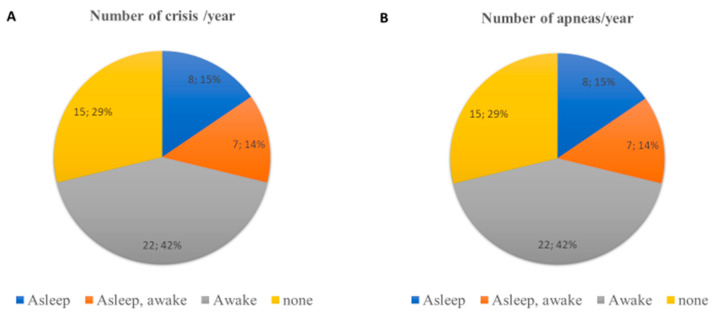
(**A**) Clinical characteristics of epileptic crisis; (**B**) clinical characteristics of apneas.

A total of 28 patients were able to walk with aids (54%), while 13 (25%) were unable to walk. The remaining 11 walked autonomously. Although case numbers are limited, a certain different prevalence of walking difficulties was noted on the basis of the disease’s clinical form (Table 3). In particular, 24% of patients with the classic form and 50% of those with the congenital variant were unable to walk. Epilepsy was very common (30/52; 57.7%), and 22 patients (42.3%) had more than 10 seizures per year (Figure 2A). As expected, 67.4% of patients with the classic form presented with epilepsy (14/43), versus 33.3% of patients with the variant with preserved language (1/3) and 50% (3/6) of the patients with the congenital variant. Apneas were present in 80.7% (42/52) of patients, with similar incidence in the various forms, more frequently while awake (42%, 22/42) (Figure 2B).

**Figure 3:**
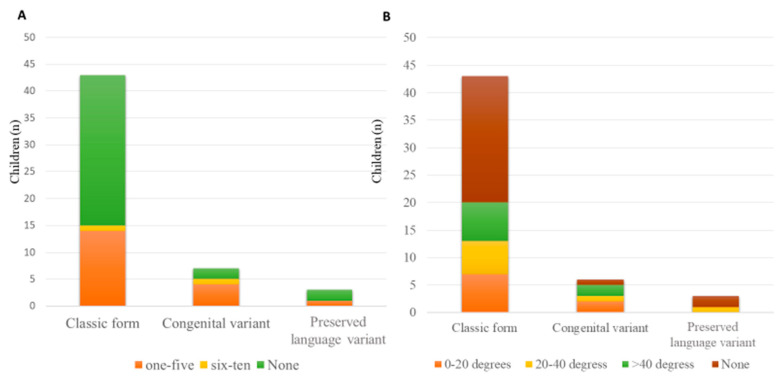
(**A**) Courses of antibiotics/year; (**B**) scoliosis and degrees of curvature.

Gastrointestinal symptoms were present in 33 patients (63.5%), with a similar frequency around 63–66% in the various forms. The most commonly reported disorders were gastro-esophageal reflux (81.4%), constipation, meteorism, and a feeling of abdominal swelling. Scoliosis was reported in 41 patients (78.8%), with a degree of curvature of 0–20 degrees in the majority of them (18 cases), while 8 patients presented a curvature of 20–40 degrees and 9 cases of more than 40 degrees (Figure 3B). No major differences were observed by disease form. In 21 (40.4%) cases, a brace was prescribed, and in 6 (11.5%) cases, corrective surgery was performed at a variable age ranging from 12 to 18 years. We noted the very low use of mechanical ventilation (1.9%), while respiratory physiotherapy was performed by only 11.5% of the patients (4 on demand, 1 periodically, and 1 continuously). In three patients, respiratory physiotherapy was performed by the therapist and in two patients by the caregivers. The respiratory physiotherapy included PEP-Mask (5 patients) and manual techniques (1 patient). The respiratory physiotherapy was advised only by four centers located in Liguria, Lazio, Sicilia, and Marche.

Considering immunization uptake, 12 patients (23%) were not fully vaccinated according to the Italian National Vaccination Schedule at the time of the survey. Furthermore, only 13/52 patients (25%) received the influenza vaccination. In several cases, it was reported that the patients had not been subjected to any vaccination; in other cases, vaccines with live attenuated viruses had not been advised. In some cases, a complete suspension of vaccinations was reported due to a presumed previous severe adverse event following the immunization.

**Figure 4:**
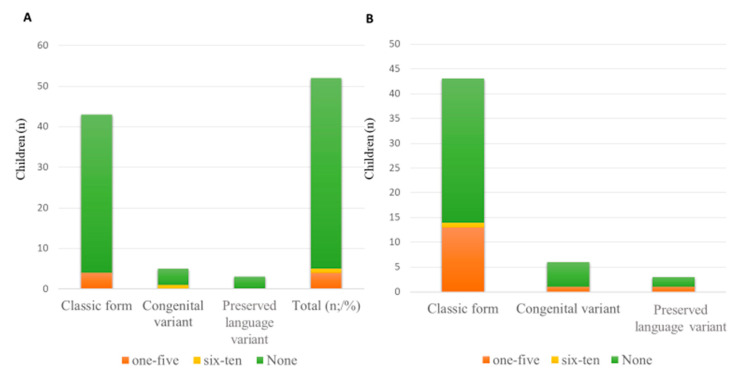
(**A**) Number of hospitalizations/year for respiratory complications; (**B**) Episodes of fever with cough/year.

Clinical characteristics and management by clinical presentation of patients are summarized in Table 3 and in Figure 2, Figure 3 and Figure 4.

## 3. Discussion

As far as we know, this is the first survey investigating the perception of caregivers regarding the management of the clinical problems of their relatives with RS. The experience of living with RS is vastly more complex than its clinical features. Therefore, this study focuses on the burden of responsibilities and level of stress as perceived by the caregivers, the need to seek assistance in several centers, and the non-homogeneous level of assistance received in different centers.

Data confirm the high complexity of this chronic, multifaceted condition, mainly characterized by the presence of epilepsy, apnea, severe scoliosis, and gastrointestinal symptoms. Therefore, patients often refer to several subspecialists in order to address their specific problems in a tailored manner.

Our data show that the patients are followed by several specialists located at a considerable distance from each other. It is well known that the healthcare mobility of children determines profound suffering due to the detachment from their place of origin, economic problems for families caused by the overall transfer cost, and work difficulties for parents/caregivers [22]. In Italy, the south-to-north mobility of every type of patient towards regional areas equipped with highly specialized healthcare structures is a common phenomenon linked to the non-homogeneous geographical distribution of healthcare resources, especially those relating to the management of complex pathologies [22]. However, in our survey, this trend was observed even for patients located in northern areas of Italy, thus suggesting a need for integrated assistance spreading all over the country.

The patients were more frequently treated by general practitioners or family pediatricians (98%) and neurologists (92%), but less frequently by psychiatrists (71%). Only 15% of the patients was followed-up by the pulmonologist, despite the fact that many of them presented with respiratory problems (apneas in 81% of patients and 2% had tracheostomy). Similarly, although 63.5% of patients complained of gastrointestinal symptoms and 2% had a gastrostomy, only 33% were followed by a gastroenterologist, and although orthopedic issues were present in 78.8% of patients, including severe scoliosis in 22% of them, only 25% were followed by the orthopedist.

These data are in contrast with the recommendations reported in a recent US Consensus Document [2].

Moreover, it is notable that 36.5% of patients had received between 1 and 5 courses of antibiotics per year, and 7.6% were hospitalized due to respiratory problems between 1 and 5 times per year. These data confirm that patients with RS are complex and fragile subjects and suggest that they are at increased risk of invasive bacterial infections or healthcare-associated infections that are commonly sustained by antimicrobial-resistant microorganisms.

Even though immunizations are mandatory, the study shows that 12 patients (23%) were not fully vaccinated according to the Italian National Vaccination Schedule at the time of the survey, and only 13 patients (25%) received the influenza vaccination. It is therefore of paramount importance to raise awareness among families and physicians about the absolute necessity of immunization.

Our data are in line with previous reports when considering the clinical presentation of the disease, as the classic RS was the most frequent form with the highest number of epilepsy cases and non-ambulatory status [9]. A recent National Survey conducted in Norway, including only adult patients, confirmed a high prevalence of six main medical issues (scoliosis, ambulation, growth, respiration, gastrointestinal dysmotility, and epilepsy) [23]^.^

However, to our knowledge, this is the first pediatric study investigating the main health issues in patients with RS and the difficulties encountered by caregivers in reaching adequate health assistance.

## 4. Limitations of the Study

The findings of this study must be considered in light of some limitations. First, our dataset was limited, and the survey was administered indirectly by email. Given the number of surveys received (52 out of 250), the results might reflect the needs of a limited target group.

As the survey was distributed via email, there may be a lower response rate, as those who chose to respond might have different experiences or views than those who did not respond.

Additionally, the study does not provide detailed demographic information about the caregivers (e.g., age, education level, socioeconomic status), which could influence their perceptions and experiences. We will add these data to a new version of the questionnaire.

In this first stage, the survey was conducted in Italian, which could exclude non-Italian-speaking caregivers from participating. As this might constitute the limit of the study, we plan to develop in the near future a questionnaire in English in order to include a larger number of participants.

It is difficult to assess whether caregivers who submitted the survey are those in a more vulnerable position and therefore willing to express their needs and frustrations, or those in a rather better situation.

Second, given the small sample size, we were unable to carry out statistical analyses by clinical subgroups of the disease. Third, a pilot study was not conducted prior to data collection. Fourth, as we did not include validated tools, future studies will be needed to better evaluate parental stress.

## 5. Conclusions

Patients with a neurodevelopmental disorder, such as RS, as well as their families, have complex needs that affect their quality of life. Therefore, both families and patients with RS must be provided with multidisciplinary health care that can identify the clinical features that most affect their quality of life. In conclusion, our data show that the majority of patients with orthopedic, respiratory, or gastroenterological disorders, representing the most frequent RS complications, were not followed by the relevant subspecialist, with the only exception of epilepsy. Therefore, it would be desirable to set up a national interdisciplinary network and develop a National Consensus Document involving multidisciplinary experts with the aim of improving the management of RS patients. Furthermore, immunization should be strongly encouraged and widespread, while raising awareness against false contraindications to vaccinations.

## Figures and Tables

**Table 1:** Characteristics of the 52 study patients.

	Patients	Range
Gender (n; %)		
Male	0 (0.0%)	
Female	52 (100.0%)	
Age at the time of the survey(years; median IQR) *	15 (7–22)	2–44
Age at diagnosis (years, median IQR)	2 (1.5–3.8)	1–13
Classic Rett syndrome (n/N; %)	43/52 (82.7%)	
Congenital variant (n/N; %)	6/52 (11.5%)	
Preserved language variant (n/N; %)	3/52 (5.8%)	

* IQR—interquartile range. Median age at the diagnosis was 2 years (IQR: 1.5–3.85; range 16 months–13 years).

**Table 2:** Specialists who followed the patients and timing of follow-ups.

Specialist	Occasionally (n/N; %)	Regularly (n/N; %)	On Demand (n/N; %)	TOTAL (n/N; %)
Neurologist/ neuropsychiatrist	9/48 (18.7%)	28/48 (58.3%)	10/48 (20.8%)	48/52 (92.3%)
Family pediatrician/ General practitioners	4/51 (7.8%)	11/51 (21.5%)	31/51 (60.7%)	51/52 (98.0%)
Physiatrist	12/37 (32.4%)	16/37 (43.2%)	9/37 (24.3%)	37/52 (71.1%)
Respiratory physiotherapist	1/6 (16.6%)	4/6 (66.6%)	1/6 (16.6%)	6/52 (11.5%)
Pulmonologist	1/8 (12.5%)	3/8 (37.5%)	4/8 (50.0%)	8/52 (15.3%)
Orthopedist	2/13 (15.3%)	3/13 (23.0%)	8/13 (61.5%)	13/52 (25.0%)
Nutritionist	2/15 (13.3%)	4/15 (26.6%)	9/15 (60.0%)	15/52 (28.8%)
Gastroenterologist	3/17 (17.6%)	5/17 (29.4%)	9/17 (52.9%)	17/52 (32.6%)
Swallow specialist	3/10 (30.0%)	1/10 (10.0%)	6/10 (60.0%)	10/52 (19.2%)
Speech therapist	0/2 (0.0%)	2/2 (100.0%)	0/2 (0.0%)	2/52 (3.8%)

**Table 3:** Clinical characteristics and management of the study patients by clinical presentation.

Symptoms	Classic Form (n/N; %)	Congenital Variant (n/N; %)	Preserved Language Variant (n/N; %)	Total (n/N; %)
Autonomous walking	10/43 (23.2%)	1/6 (16.6%)	0/3 (0.0%)	11/52 (21.1%)
Walking with aids	22/43 (51.1%)	2/6 (33.3%)	3/3 (100.0%)	28/52 (53.8%)
Non-ambulatory status	10/43 (23.2%)	3/52 (5.7%)	0/3 (0.0%)	13/52 (25.0%)
Epilepsy	14/43 (32.5%)	3/6 (50.0%)	1/3 (33.3%)	30/52 (57.6%)
Apneas	34/43 (79.0%)	5/6 (83.3%)	3/3 (100.0%)	42/52 (80.7%)
Polypneas	10/43 (23.2%)	1/6 (16.6%)	1/3 (33.3%)	12/52 (23.0%)
Gastrointestinal issues	27/43 (62.7%)	4/6 (66.6%)	2/3 (66.6%)	33/52 (63.5%)
Dysphagia	15/43 (34.8%)	3/6 (50.0%)	2/3 (66.6%)	20/52 (38.4%)
Scoliosis	34/43 (79.0%)	4/6 (66.6%)	3/3 (100.0%)	41/52 (78.8%)
Tracheostomy	0/43 (0.0%)	1/6 (16.6%)	0/3 (0.0%)	1/52 (1.9%)
Percutaneous endoscopic gastrostomy (PEG)	0/43 (0.0%)	1/6 (16.6%)	0/3 (0.0%)	1/52 (1.9%)
Invasive mechanical ventilation	0/43 (0.0%)	1/6 (16.6%)	0/3 (0.0%)	1/52 (1.9%)
Intensive care unit admissions	7/43(16.2%	1/6 (16.6%)	0/3 (0.0%)	8/52 (15.3%)
Immunizations	9/43 (20.9%)	3/6 (50.0%)	0/3 (0.0%)	23/52 (44.2%)
Influenza vaccination	10/43 (23.2%)	1/6 (16.6%)	2/3 (66.6%)	13/52 (25.0%)
Chest physio- therapy	4/43 (9.3%)	2/6 (33.3%)	0/3 (0.0%)	6/52 (11.5%)

## Data Availability

The datasets generated and analyzed during the current study are not publicly available due to privacy reasons but are available from the corresponding authors upon reasonable request in an anonymous form.

## Appendix A

Questionnaire for caregivers.

**Table A1:** Survey to improve respiratory disorders in Rett syndrome.

Child’s initials	
Current age/ Date of birth	
Age at diagnosis	
Genetics	MECP2 yes no If no, specify the mutation
Referral center	
Therapy	

Who are the professionals who follow your daughter?

**Table A2:** Specialists who followed the patients and timing of follow-ups.

Professional Figures	Yes/No	If Yes, How Often	
Neurologist/neuropsychiatrist	yes no	Regularly	Occasionally
Family pediatrician/General practitioners	yes no	Regularly	Occasionally
Physiatrist	yes no	Regularly	Occasionally
Respiratory physiotherapist	yes no	Regularly	Occasionally
Pulmonologist	yes no	Regularly	Occasionally
Orthopedist	yes no	Regularly	Occasionally
Nutritionist	yes no	Regularly	Occasionally
Gastroenterologist	yes no	Regularly	Occasionally
Swallow specialist	yes no	Regularly	Occasionally
Other: (specify)	yes no	Regularly	Occasionally

Presents a:  Classic form□    Congenital variant□   Mild learning disabilities□

Autonomous walking  yes□ no□ with aids□   Non-ambulatory status □

Epilepsy  yes □  no □   If yes, crisis/year 1–5 □  6–10 □  >10 □

Tachypnea yes □ no □ Apneas yes□ no□ if yes, asleep □ awake □

Gastrointestinal issues: yes □ no □,  if yes, gastro-esophageal reflux, RGE □    

Constipation □    meteorism and a feeling of abdominal swelling □  other ……………………………….

Dysphagia: yes □ no □

Scoliosis? yes□ no□  if yes, possibly specify degrees of curvature 0–20□ 20–40 □ > 40 □

Has a corset been prescribed? yes □ no □ Has she undergone surgery? yes □ no □ if yes, specify at what age.....

Tracheostomy? yes □ no □ if yes, specify at what age.....

Mechanical ventilation? Yes □ no □ if yes, night-time □ H24 □

Percutaneous endoscopic gastrostomy (PEG)? yes□ age … no □ other ….

Surgery for RGE?   yes age… no □ by Nissen yes □ age … no □

Intensive care unit admissions? yes □ no □ if yes, how many? ………

Does the patient comply with the Italian National Vaccination Schedule?

yes  no

If no why?

……………………………………………………………………..

Influenza vaccination? yes    no

How many times has she been hospitalized for respiratory problems in the last 12 months?

1–5  5–10  > 10    

How many times fever with cough in the last 12 months?

1–5  5–10  > 10    

How many cycles of antibiotics in the last year?

1–5  5–10  > 10    

Respiratory physiotherapist? yes    no  If yes, in which cent was it prescribed?

if yes, regularly?   cyclically   on demand

With the physiotherapist? yes  no

performed by the parent/caregiver? yes   no

use:     Cough machine     yes   no

       Pep-Mask       yes   no

Manual techniques  yes   no

       Other ……………………………………………

Notes (kindly report anything else you deem useful)
